# Actual vs. Perceived Competency Development—How Can Virtual Patients Impact Pharmacist Pre-Registration Training?

**DOI:** 10.3390/pharmacy8030138

**Published:** 2020-08-05

**Authors:** Jessica Thompson, Simon White, Stephen Chapman

**Affiliations:** School of Pharmacy and Bioengineering, Keele University, Keele, Newcastle ST5 5BG, UK; s.j.white@keele.ac.uk (S.W.); s.r.chapman@keele.ac.uk (S.C.)

**Keywords:** virtual patients, high fidelity simulation training, pharmacy education, computer-based education, pharmacy practice, online learning

## Abstract

Virtual patients are an active learning pedagogical tool that simulate clinical scenarios. There is an established disparity in pre-registration examination pass rates depending on whether individuals complete their training in a community or hospital pharmacy. This study aimed to evaluate virtual patient (VP) and non-interactive (NI) case studies, concerning knowledge, skill and confidence development of pre-registration pharmacist trainees. A quasi-experimental evaluation was conducted. Pre-registration pharmacists completed three VP or NI case studies. Each case study was associated with a pre-and post-knowledge quiz. Pre-registration trainees were invited to complete a questionnaire consisting of Likert ranking statements and open-ended questions on the case study features, usability and individual development. Both learning tools significantly improved trainees’ knowledge on the topic areas (except for the NI group in case study 3). Although no significant differences in knowledge improvement were identified between the learning tools, trainees who used the VP reported the development of a wider knowledge base and skill set, an increase in confidence for practice and an opportunity to apply their learning. The sector in which pre-registration pharmacists were completing their training (community or hospital) had a significant impact on knowledge improvement in the three case studies. Future research evaluating VPs with pre-registration and qualified pharmacists should be conducted to explore their benefits and establish their effectiveness as learning tools.

## 1. Introduction

A four-year undergraduate Master of Pharmacy (MPharm) degree and a work-based pre-registration training year are required to qualify as a pharmacist in the United Kingdom (UK). The experiences of each individual during their pre-registration training year will vary substantially due to factors such as the individual’s motivation, the support of the pre-registration tutor and the experiences had. This has led to a disparity in pre-registration examination pass rates [1], particularly between those who complete their training in a hospital versus a community pharmacy. It is evident that training needs to adapt to ensure that individuals are adequately supported to provide safe and effective patient-centred care, especially with the advancing pharmacist roles, irrespective of the sector in which they complete pre-registration training [2].

A range of simulation tools exist and have been written about in healthcare literature [3,4,5]. This paper will focus on virtual patients (VPs). VPs can be defined as ‘a specific type of computer-based program that simulates real-life clinical scenarios; learners emulate the roles of health care providers to obtain a history, conduct a physical exam, and make diagnostic and therapeutic decisions’ [6]. Unlike other forms of computer assisted learning, the outcomes of VP simulations are directly related to learner input, which has found to encourage users to take responsibility for their actions and learn from their mistakes [7]. VPs can take different forms, with varying fidelities. They should be designed to be realistic but not to the detriment of falling into the “uncanny valley”, which occurs when the degree of visual similarity to real humans becomes unsettling and can trigger negative thoughts [8]. Traditional learning tools allow individuals to learn in a linear fashion whereas VPs allow learners to access real-world scenarios and make mistakes, promoting a more dynamic learning approach which may result in better retention of knowledge and skills [9]. Providing a safe environment to practice, make mistakes and visualise the consequences of their actions may encourage reflective learning and prevent errors in the future [10]. Previous research illustrates the benefit that simulation has on reducing the pressure and anxiety felt by individuals when completing new tasks [3]. This may translate into increased confidence in practice.

The majority of research evaluating VPs has been conducted in medicine or nursing [7,11,12]. Simulation tools can be classified based on their fidelity; all types and fidelities of simulation can be effective learning tools depending on the intended learning outcomes [5]. Those classified as higher fidelity have been found to promote development of emotional intelligence, communication skills, clinical reasoning skills and knowledge on a range of clinical conditions [11,12,13]. Less research has been conducted into the utilisation of VPs in undergraduate or postgraduate pharmacist education and training, and only a few of these studies have evaluated higher fidelity simulations [14,15,16,17,18,19,20,21,22,23]. The majority of this work has been undertaken in America which may be due to the recognition of the benefits which simulation can offer, as per The Accreditation Council for Pharmacy Education standards [24]. Only one study, focused on knowledge and confidence development of delivering a single community pharmacy service, has evaluated VP use in pre-registration pharmacist training [25]. This is a common trend in the literature, with research tending to focus on a single learning outcome and rarely comparing against other learning tools. Whilst this may be difficult, as noted by Cook et al. (2010), the potential benefits that VPs can bring to pharmacist education requires further evaluations to establish them as effective learning tools [11].

The aim of this research was to evaluate virtual patient and non-interactive case studies, with respect to knowledge, skill and confidence development of pre-registration pharmacist trainees. 

## 2. Materials and Methods 

**Figure 1:**
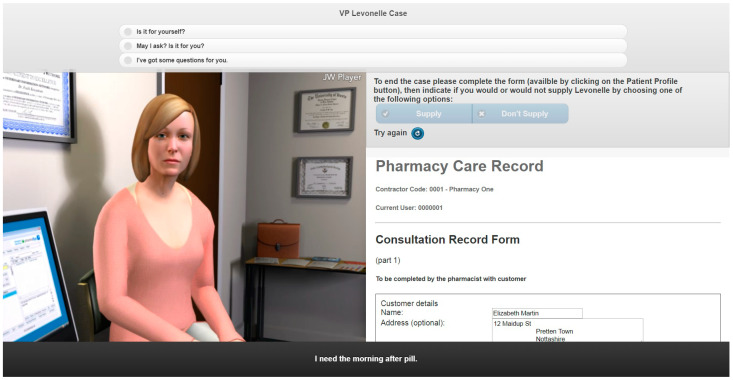
Screenshot of the emergency hormonal contraception (EHC) virtual patient (VP) simulation.

**Figure 2:**
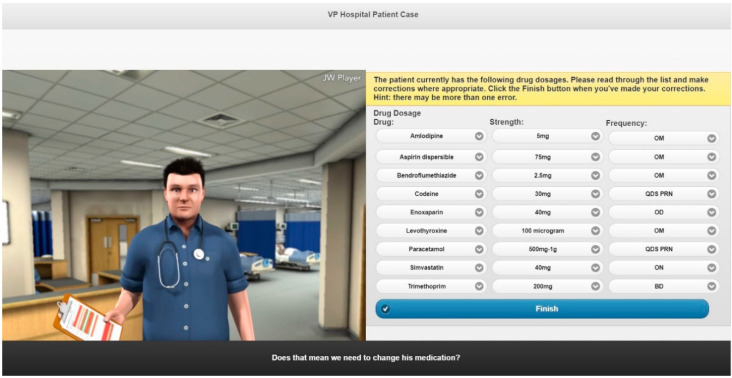
Screenshot of the renal function VP simulation.

**Figure 3:**
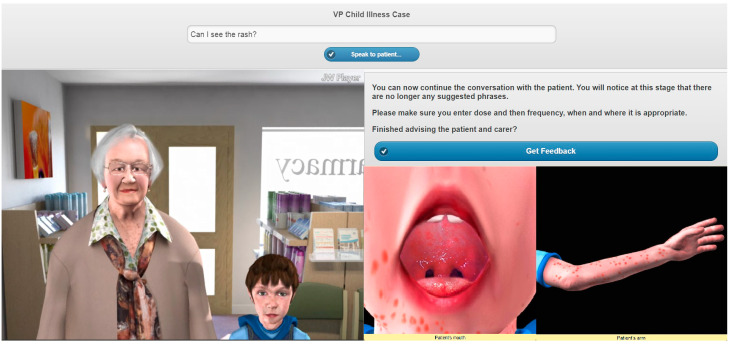
Screenshot of the childhood illnesses VP simulation.

Three VP case studies were created on the topics of: (1) emergency hormonal contraception (EHC), (2) calculation of renal function and (3) childhood illnesses (Figure 1, Figure 2 and Figure 3) [26]. A walkthrough of the EHC case can be found in the Supplementary Materials Video S1.

These topics were identified from a review of the literature, the aim of the study and conversations between the research team and first year qualified pharmacists, and included areas which trainees may find it difficult to show their competence in due to training variations. The clinical elements of the cases were based on relevant guidelines and resources which pharmacists use in everyday practice. Intended learning outcomes for each VP simulation were established. The script for each VP simulation was created in consultation with two registered pharmacists with the relevant expertise. The research team worked with the Digital Development Team from the School of Pharmacy and Bioengineering at Keele University to design the simulations. Learners could interact with the VP via multiple-choice input, free-text input or clicking relevant objects. All VPs in this study were designed to express humanistic characteristics through simple body language, movements and pre-recorded voice replies; a textual response is also displayed on the screen. The VP was animated to provide spoken feedback at the end of each simulation, and this was also displayed in a textual format. Each VP utilised computer-generated animated videos via HTML, CSS and JavaScript resource accessing video renders. The simulations were stored online ensuring ease of access for all users [27]. 

Three non-interactive (NI) case studies were also created on the same three topics. These existed as Google Forms. Each case study consisted of the intended learning outcomes, an introductory paragraph and free-text questions. Each question required an answer. Upon submitting, feedback was provided by displaying the “model answer” for each question, an explanation of the correct answer and signposting for further reading. Although trainees had to input their answers, there was no two-way interaction. The NI case studies can be found in the Supplementary Materials File 2.

All participants provided their informed consent for inclusion before they participated in the study. The protocol was approved by the Ethics Committee of Keele University (ERP2221). A quasi-experimental evaluation with a non-equivalent control group design was carried out. This requires data to be collected from two groups at pre-test, followed by an intervention and a second data collection at post-test [28]. Pre-registration pharmacist trainees completing their training in a UK-based hospital or community pharmacy (training year 2014–2015) were recruited by sending emails to those in their final year of study at Keele University School of Pharmacy, presentations at regional hospital training days and emails to those undertaking their training with a large community pharmacy chain. The required sample size to obtain statistically significant results was calculated to be 52, based on a *p*-value of 0.05, a power level of 0.8 and an effect size of 0.8. Pre-registration trainees were non-randomly assigned to receive the VP simulations or NI case studies. Each case study had a pre-and post-quiz consisting of 20 multiple choice questions (MCQs) on that topic area (same questions used pre-and-post) to assess knowledge changes. Each case study was made available for one month, during which time it could be completed as many times as required. Participants were required to complete the pre-MCQs before they could access the case and were unable to access the next case study without completing the post-MCQs within the one-month period. Upon completion of the three case studies, perceptions on the learning tools were gathered using a pre-validated questionnaire consisting of Likert-ranking statements (1 = strongly disagree, 5 = strongly agree) and open-ended questions [27]. The pre–post MCQs and data collection questionnaire can be seen in the Supplementary Materials Files 3 and 4, respectively. Participants were then provided with access to the other type of case study to use at their own leisure as an extra learning tool prior to their pre-registration examination.

Answers on the MCQs were marked as being “correct” or “incorrect”. Data were initially screened for completeness and violation of assumptions. Pre-registration trainees who did not complete each set of pre-and post-MCQs were excluded from further analysis. Data were analysed using SPSS software. The normality of data distribution was assessed using the Shapiro–Wilk test and Q–Q plots, which resulted in parametric analysis of the MCQ scores via dependent and independent t-tests. P values of less than 0.05 were reported as statistically significant. Quantitative data from the Likert scales were analysed descriptively and non-parametrically via Mann–Whitney U tests. Qualitative data from the open-ended questions were subject to content analysis [29]. All comments provided by participants were imported into a Microsoft Excel spreadsheet for coding. Comments were received on a range of issues, which led to a more inductive approach initially to ensure all relevant comments were included in the analysis, before comments were categorised into themes deductively. The content analysis process involved the identification, organisation and indexing of comments to provide frequencies of coded themes [30]. Cronbach’s alpha was used for reliability analysis of internal consistency.

## 3. Results

### 3.1. Demographic Results

One hundred sixty five pre-registration trainees from the UK consented to participate. The majority of participants were female (71.5%), in their early twenties (mean age of 23) and of White British (43%) or Asian Indian (14%) ethnicity. Participants had studied their MPharm degree at one of the 26 UK-based universities accredited by the General Pharmaceutical Council (GPhC) to offer an MPharm degree. The majority of participants (69%) reported no previous VP experience. More than half of the participants (56%) were undertaking their training in community pharmacy.

A decline in the response rate for each case study was observed (Table 1). The overall response rate for the questionnaire was 34% but, as only those individuals who completed the third case study were invited to complete the questionnaire, an actual response rate of 89% was obtained.

### 3.2. Pre–Post MCQ Results

Table 2 displays the statistical analysis of the pre- and-post MCQs. Knowledge improvement was determined by the number of questions answered correctly on the post-MCQs compared to the pre-MCQs. For all three case studies, trainees in both groups answered significantly more questions correctly on the post-MCQs than at baseline (except for the NI group in case 3), which can be seen in columns one and two. No significant differences between the VP and NI trainees’ knowledge improvement was identified for any case study (column 3). In contrast, significant improvements in knowledge were found between the training sectors (column 4). Case study one (EHC) improved hospital-based trainees’ knowledge more significantly, whereas case studies two (renal function) and three (childhood illnesses) improved community-based trainees’ knowledge more significantly.

### 3.3. Likert Scale Analysis

A 20-item questionnaire covering the themes of case study features, usability and individual development was used (1 = strongly disagree, 5 = strongly agree). The questionnaire was associated with very high reliability based on a Cronbach-α score of 0.95. Analysis of the findings illustrated no significant differences between the median VP scores and median NI scores for any of the statements based on the Mann–Whitney U test (Table 3). Higher agreement scores from trainees in the VP group were associated with Likert statements that related to the realism of the case studies and trainees’ interest and enjoyment in completing them. Additionally, trainees agreed more that they were developing skills and knowledge for practice, including more complex skills such as clinical reasoning. The VP simulations also led to trainees agreeing to a greater extent that they felt confident for practice and were taking responsibility for their learning. Despite this, the proportion of trainees disagreeing that they were developing skills, improving confidence for the exam and real practice and collaborating with other healthcare professionals was also higher for the VP group. The NI group tended to have a high proportion of trainees who were “undecided” with these statements. Trainees who used the NI case studies reported higher agreement that the objectives of the case studies were easier to understand, they were easier to access and individuals reported improved confidence for the pre-registration examination.

### 3.4. Qualitative Analysis

The open-ended questions on the questionnaire covered: trainees likes and dislikes towards the case studies, improvements that could be made to the case studies and how the case studies could be utilised within the pre-registration training year. From the content analysis, four main topics were derived: features of the learning tools; ease of use of the learning tools; trainee development from use of the learning tools; and integration of the learning tools into training. Themes and frequencies from the content analysis can be found in the Supplementary Materials File 5.

#### 3.4.1. Features of the Learning Tools

Pre-registration trainees commented on the realism of the learning tools, including the relevance of topics to practice, the novelty and interactivity of the case studies and the level of feedback provided. All trainees who used the VP reported it as being novel; this was not reported from those who completed the NI cases. Almost all of the trainees who used the VP commented on its realism, reporting that ‘the actual simulation of the patient in terms of the voice and movement was really good…very realistic.’ No comments were received on the realism of the NI learning tool, however the majority of individuals in each group reported the topics as being “real-to-life” which helped them to focus on what knowledge and skills they needed for their future careers. The interactivity of the VP technology was reported as improving its realism as trainees could ‘ask questions to a patient and they responded like a real-life consultation’, encouraging them to consider the language and phrases they were using. Comments were received from those who used the NI case studies, however, these were all associated with the lack of interactivity and the belief that ‘[more] stimulation is required to prepare for real life situations.’ The provision of immediate feedback was reported as being beneficial to learning by the majority of trainees; those in the VP group reported that the personalised feedback was particularly useful.

#### 3.4.2. Ease of Use of the Learning Tools

The majority of participants who completed the VP case studies reported one or more technology-based issues. The most common surrounded the recognition of the free-text software. One trainee in particular reported that ‘more options for what we could ask the patient would have helped us more easily obtain the information we needed.’ In contrast, whilst trainees reported frustration from the recognition problems, they also used it as an opportunity to broaden their knowledge and re-phrase questions. Trainees from the VP group also reported on struggling with accessibility when using certain interfaces or software. This was easily solved by recommending they try an alternative Internet browser but did ‘[make] it difficult and inconvenient’. The users of the NI case studies reported them as easily accessible.

#### 3.4.3. Trainee Development

Both types of case study were reported to develop pre-registration trainees’ knowledge, skills and confidence levels. Those trainees in the VP group reported the development of ‘clinical responsibility and practice at decision making’ as well as improving communication skills through the use of free-text input. When trainees from the NI group reported on skill development, they focused on their academic literacy and calculation abilities. More trainees in the VP group reported knowledge development, particularly related to completing the cases, whereas those from the NI group reported the pre–post quizzes were more effective at enhancing their knowledge. In addition, trainees from the VP group reported that the simulations provided them with a ‘chance to practice in a safe environment’ and that they were able to apply their learning to practice to ‘reinforce [their] knowledge.’

#### 3.4.4. Integration of the Learning Tools

The majority of pre-registration trainees reported using either type of case study as an individual learning tool. They reported that they gave ‘more structure to learning and revision.’ Trainees from both groups also reported that the case studies could fit into a group learning setting, such as pre-registration training study days. Those who completed the VP simulations offered thoughts on their use to develop teamwork and support interdisciplinary learning. A number of trainees from the VP group reported using the simulations to aid their competency development and as evidence for having met training requirements. They reported that using these tools in a similar way once qualified would be helpful for continuing professional development (CPD). Trainees from the VP group also reported that the simulations would be useful for training in other sectors of pharmacy, both during pre-registration training to ‘help with cross-sector placements…and those who don’t get much patient facing experience’ and in the future if transitioning roles.

## 4. Discussion

The aim of this study was to evaluate VP and NI case studies, with respect to knowledge, skill and confidence development of pre-registration pharmacist trainees. Both learning tools significantly improved trainees’ knowledge on the topic areas (except for the NI group in case 3). This may not be a surprise, as providing either learning tool was above standard training. Although no significant differences in knowledge improvement were identified between the learning tools, there was difference in trainees’ perception of their development. Trainees who used the VP reported the development of a wider knowledge base and skillset, an increase in confidence for practice and an opportunity to apply their learning, whereas for the NI cases there was more focus on technical issues such as knowledge acquisition and numeracy. VP technology may be of greater benefit in allowing the application of knowledge and skills rather than their specific development. A significant finding from this research was the difference in knowledge improvement between the sectors. This may indicate that, perhaps, as long as individuals are pro-active in their learning, the learning tool itself is not important and what is more important is ensuring the topics are relevant to user needs. Case study one (EHC) improved hospital-based trainees’ knowledge more significantly, whereas case studies two (renal function) and three (childhood illnesses) improved community-based trainees’ knowledge more significantly. Case studies one and two improved trainees’ knowledge most significantly in the sector where they were unlikely to have this experience. Case study three improved community-based trainee’s knowledge to a greater extent. Childhood conditions may be an area which trainees in either sector had limited experience with due to the specialist nature of paediatrics. Community-based trainees may be more likely to come across the childhood conditions included in this case study as they were considered more minor ailment-based conditions requiring over-the-counter treatment or self-care advice, thus community-based trainees may have found the case study more useful as a preparatory tool for practice.

The role of a pharmacist in the UK is transitioning from just compounding and dispensing of medicines into a more clinical, patient-centred role [31]. With this, opportunities to work in different sectors has also arisen, such as within general practice and wider multidisciplinary teams [32]. This requires pharmacists to be confident and assertive in settings they may not be used to. Pharmacists have been identified as being less confident than other healthcare professionals, which may be due to the reduced clinical exposure had at an undergraduate level [33]. Simulation-based learning can be instrumental in promoting experiential situated learning and allowing demonstration of the higher levels of Miller’s triangle [34,35,36]. This was evidenced in this study, as pre-registration trainees reported that the VP provided an opportunity for them to apply their learning. Some trainees expressed the use of the simulations to demonstrate their competence, which they may otherwise have struggled with due to training sector variability. This was not reported from those who used the NI case studies. Providing resources which encourage active learning may be beneficial to bridge the established gap in variation between the training sectors and provide an opportunity to upskill pharmacists for the wider roles.

The realism of the VP was expressed as a positive feature of the simulations. Pre-registration trainees reported that the interactivity of the VP simulations increased their realism and allowed them to feel more immersed within the case studies and really feel like the pharmacist in the scenario. In contrast, the lack of interactivity of the NI cases was reported by the pre-registration trainees to affect their ability to apply their learning from the case studies to real-life practice. VPs provide a safe environment for users to make mistakes and learn from them, without risking harm to a real patient [37]. Completing either type of case study led to pre-registration trainees reportedly feeling more confident for the pre-registration examination, however, a greater number of trainees who used the VPs reported themselves as being confident about caring for real patients. This may be due to the increased application of knowledge that VP simulations promote [7]. Learning techniques which require users to actively engage in the process support higher-order learning outcomes and have been found to encourage independent learning [38]. The requirements of continuing professional development mean this is an essential skill for a pharmacist. Pre-registration trainees who used the VP reported feeling greater responsibility for their learning, which may be a result of trainees’ actions and decisions affecting simulation outcomes and the individualised feedback potentially invoking reflective practice.

Pre-registration trainees who completed either type of case study reported that their knowledge had improved and they were developing skills for future practice. Although no statistically significant results were obtained to reinforce this finding, individual’s thoughts on the usefulness of a learning tool are important contributors to their overall utilisation. Pre-registration trainees reported the development of real-life complex skills and knowledge from completing the VP simulations themselves, whereas those who completed the NI case studies reported knowledge development from the pre–post quizzes and more simple skill development—which have previously been shown to be developed successfully from “rote learning” [39]. This advocates the use of the appropriate learning tools at the necessary level of training and for the appropriate learning outcomes. It has been identified that VPs may add little value when learners are at the lower levels of Bloom’s taxonomy [40]; instead, they may provide most benefit when knowledge and skills are combined and applied in a problem-solving context, particularly when direct patient contact is not possible [12]. VPs may therefore be especially useful for pre-registration trainees and qualified pharmacists as they can be created to provide a wide variety of simulations for the different training or work sectors.

Previous research in medicine has explored the underutilisation of VPs despite the technology being available [41]. Similar research has not been conducted in pharmacy schools. The study of VPs in pharmacy education and training is less than in other healthcare professions [7,12,15] and there is only one other evaluation of VPs in UK pre-registration pharmacist training [18]. It was identified in 2012 that more research should be invested in developing virtual patient technology specifically for teaching at a graduate level [14]. This may still be limited due to the multiple designs and technologies that exist for VPs, which makes it difficult for researchers to ascertain their effectiveness and therefore adopt “best practice”.

A potential limitation of this study was the participant response rate. However, this is in line with other research that utilised prolonged testing periods with multiple data collection components, and the required sample size to calculate statistical significance was achieved [42]. Ethical approval was not requested to follow-up the participants who dropped out of the research, but would be useful for future work. The profile of respondents from this quasi-experimental evaluation was diverse and consistent with the distribution of pharmacists in practice [43]. This study investigated actual knowledge improvement and self-reported development; there was no assessment of competence in practice which may be a focus for future research. The same questions were used on the pre- and post-quizzes and participants could have checked resources during this time period. To minimise this, participants were not made aware that they would be asked the same questions on the post-quiz.

There are many areas for future research. Exploring the benefits that simulation can bring to training sector variation would expand on the findings from this research. It would be especially useful to evaluate VPs as a learning tool with practicing pharmacists to determine the benefits they may provide. Conducting research which not only evaluates competence in practice, but the lasting effects of knowledge improvement after VP application would also add to the current literature.

## 5. Conclusions

Both types of case study were effective at improving pre-registration pharmacists’ knowledge, especially when simulating experiences that they may not have due to training sector variation. Virtual patient simulations were also associated with the self-reported development of more complex skills and self-confidence for practice. The application of knowledge is a benefit of virtual patients, and their use in graduate and qualified pharmacists should be further explored.

## Figures and Tables

**Table 1:** Response rates (RR) for each case study.

Stage of Research	No. of Participants (% RR)		
	VP Group	NI Group	Overall
Consented	83	82	165
Case 1 Completion	60 (72%)	66 (80%)	126 (76%)
Case 2 Completion	42 (51%)	47 (57%)	89 (54%)
Case 3 Completion	27 (33%)	36 (44%)	63 (38%)
Questionnaire Completion	24 (29%)	32 (39%)	56 (34%)

**Table 2:** Knowledge improvement scores pre–post.

Case Study	Mean (SD) Knowledge Improvement VP Group (Pre–Post)	Mean (SD) Knowledge Improvement NI Group (Pre–Post)	Independent *t*-Test VP vs. NI Knowledge Improvement	Independent *t*-Test Hospital vs. Community Knowledge Improvement
1	2.18 (1.92) ^a^	2.11 (1.96) ^a^	t _(100)_ = 0.183	t _(72)_ = 1.898 ^b^
2	1.37 (1.67) ^b^	1.54 (1.63) ^b^	t _(63)_ = −0.429	t _(63)_ = −0.249 ^a^
3	1.17 (1.44) ^b^	1.24 (2.21)	t _(46)_ = −1.665	t _(46)_ = −1.824 ^b^

Significance: ^a^ (*p* < 0.001), ^b^ (*p* ≤ 0.05).

**Table 3:** Likert statements with their associated median, interquartile range (IQR) and percentage agreement scores.

Likert Statement	VP Median Score (IQR)	NI Median Score (IQR)	Percentage Agree/Strongly Agree		Percentage Disagree/Strongly Disagree	
			VP	NI	VP	NI
The case studies provided a realistic patient simulation	4 (3–4)	3 (3–4)	58.3%	46.9%	4.2%	12.5%
When completing the case studies I felt as if I were the pharmacist caring for this patient	4 (3–5)	4 (3–4)	66.7%	53.1%	16.7%	15.6%
When completing the case studies I felt I had to make the same decisions as a pharmacist would in real life	4 (4–5)	4 (3–5)	79.2%	56.3%	8.3%	6.3%
The case studies were interesting	4 (4–5)	4 (3–5)	83.3%	68.8%	0%	6.3%
The case studies were enjoyable	4 (4–5)	4 (3–4)	79.2%	62.5%	8.3%	9.4%
The difficulty of the case studies were appropriate for my level of training	4 (4–5)	4 (4–5)	87.5%	78.1%	8.3%	6.3%
The feedback I received was adequate for my needs	4 (3–4.25)	4 (3–4.25)	66.7%	68.8%	8.3%	9.4%
The objectives for the case studies were clear and easy to understand	4 (3–4.25)	4 (4–5)	62.5%	78.1%	16.7%	6.3%
I was able to access the case studies at my convenience	4.5 (3.75–5)	4.5 (4–5)	75%	78.1%	8.3%	3.1%
The case studies helped develop my clinical reasoning skills	4 (4–5)	4 (3–4)	79.2%	65.6%	16.7%	6.3%
The case studies helped develop my problem-solving and decision-making skills	4 (3–5)	4 (3–4)	66.7%	65.6%	12.5%	6.3%
The case studies have helped me to put theory into practice	4 (3.75–5)	4 (3–5)	75%	56.3%	8.3%	6.3%
I am confident I am developing skills from the case studies that will be required in practice	4 (3–4)	4 (3–5)	66.7%	59.4%	12.5%	6.3%
I am confident I am gaining knowledge from the case studies that will be required in practice	4 (4–5)	4 (3–5)	87.5%	65.6%	0%	6.3%
It is my responsibility to learn what I need to know from the case studies	5 (4–5)	4.5 (3.75–5)	91.7%	75%	0%	3.1%
Completing the case studies has improved my confidence for the pre-registration exam	3 (3–4)	3 (3–4)	45.83%	46.9%	16.7%	15.6%
I feel better prepared to care for real-life patients	4 (3–4)	3 (3–4)	62.5%	40.6%	16.7%	12.5%
I feel more confident about collaborating with patients and other healthcare professionals	3 (2–4)	3 (3–4)	41.7%	34.4%	37.4%	18.8%
The case studies have increased my confidence about practicing as a pharmacist	3 (3–4)	3 (3–4)	45.8%	43.8%	16.7%	6.3%
Overall, the experience has enhanced my learning	4 (3–5)	4 (4–5)	70.8%	78.1%	8.3%	3.1%

All results were non-significant based on the Mann–Whitney U test (*p* > 0.05). An “undecided” option was provided; the percentage agreement for this selection is not expressed in the table.

## Supplementary Materials

The following are available online at https://www.mdpi.com/2226-4787/8/3/138/s1, Video S1: EHC VP walkthrough, Supplementary Materials File 2: NI case studies, Supplementary Materials File 3: Pre–post MCQs, Supplementary Materials File 4: Data collection questionnaire, Supplementary Materials File 5: Content analysis.

## References

[1] Blenkinsopp A., Marshall K., Roberts G., Wisher S., McNair K. (2015). General Pharmaceutical Council Survey of 2013/2014 Pre-Registration Trainees.

[2] John C. (2018). The changing role of the pharmacists in the 21st century. Pharm. J..

[3] Ker J., Bradley P., Swanwick T. (2010). Simulation in medical education. Understanding Medical Education: Evidence, Theory and Practice.

[4] Rosen K.R. (2008). The history of medical simulation. J. Crit. Care.

[5] Seropian M.A., Brown K., Gavilanes J.S., Driggers B. (2004). Simulation: Not just a manikin. J. Nurs. Educ..

[6] Association of American Medical Colleges (AAMC) (2007). Effective Use of Educational Technology in Medical Education. Colloquium on Educational Technology: Recommendations and Guideline for Medical Educators. https://store.aamc.org/downloadable/download/sample/sample_id/111/.

[7] Cook D.A., Triola M.M. (2009). Virtual patients: A critical literature review and proposed next steps. Med. Educ..

[8] Mori M., Macdorman K.F., Kageki N. (2012). The uncanny valley [from the field]. IEEE Robot Autom. Mag..

[9] Poulton T., Ellaway R.H., Round J., Jivram T., Kavia S., Hilton S. (2014). Exploring the efficacy of replacing linear paper-based patient cases in problem-based learning with dynamic web-based virtual patients: Randomized controlled trial. J. Med. Internet Res..

[10] Ellaway R.H., Poulton T., Smothers V., Greene P. (2009). Virtual patients come of age. Med. Teach..

[11] Cook D.A., Erwin P.J., Triola M.M. (2010). Computerized virtual patients in health professions education: A systematic review and meta-analysis. Acad. Med..

[12] Kononowicz A.A., Woodham L.A., Edelbring S., Stathakarou N., Davies D., Saxena N., Car L.T., Carlstedt-Duke J., Car J., Zary N. (2019). Virtual patient simulations in health professions education: Systematic review and meta-analysis by the Digital Health Education Collaboration. J. Med. Internet Res..

[13] Issenberg S.B., McGaghie W.C., Petrusa E.R., Lee G.D., Scalese R.J. (2005). Features and uses of high-fidelity medical simulations that lead to effective learning: A BEME systematic review. Med. Teach..

[14] Jabbur-lopes M.O., Mesquita A.R., Silva L.M., De Almeida Neto A.D., Lyra D.P. (2012). Virtual patients in pharmacy education. Am. J. Pharm. Educ..

[15] Richardson C.L., White S., Chapman S. (2019). Virtual patient technology to educate pharmacists and pharmacy students on patient communication: A systematic review. BMJ Simul. Technol. Enhanc. Learn..

[16] Thompson J.F., White S., Chapman S. (2016). An evaluation into the effectiveness of virtual patients compared with non-interactive learning techniques in pre-registration training. Int. J. Pharm. Pract..

[17] Thompson J.F., White S., Chapman S. (2019). Can interactive clinical avatars improve pre-registration pharmacists’ knowledge base?. Int. J. Pharm. Pract..

[18] Taglieri C.A., Crosby S.J., Zimmerman K., Schneider T., Patel D.K. (2017). Evaluation of the use of a virtual patient on student competence and confidence in performing simulated clinic visits. Am. J. Pharm. Educ..

[19] McDowell J., Styles K., Sewell K., Trinder P., Marriott J., Maher S., Naidu S. (2016). A simulated learning environment for teaching medicine dispensing skills. Am. J. Pharm. Educ..

[20] Ferrone M., Kebodeaux C., Fitzgerald J., Holle L. (2017). Implementation of a virtual dispensing simulator to support US pharmacy education. Curr. Pharm. Teach. Learn.

[21] Cláudio A.P., Carmo M.B., Pinto V., Cavaco A. (2015). Virtual humans for training and assessment of self-medication consultation skills in pharmacy students. Proceedings of the 10th International Conference on Computer Science & Education (ICCSE 2015), Cambridge, UK, 22–24 July 2015.

[22] Bindoff I., Ling T., Bereznicki L., Westbury J., Chalmers L., Peterson G., Ollington R. (2014). A computer simulation of community pharmacy practice for educational use. Am. J. Pharm. Educ..

[23] McFalls M. (2013). Integration of problem-based learning and innovative technology into a self-care course. Am. J. Pharm. Educ..

[24] The Accreditation Council for Pharmacy Education (ACPE) (2015). Accreditation Standards and Key Elements for the Professional Program in Pharmacy Leading to the Doctor of Pharmacy Degree.

[25] Zlotos L., Power A., Hill D., Chapman P. (2016). A scenario-based virtual patient program to support substance misuse education. Am. J. Pharm. Educ..

[26] Thompson J. (2018). Clinical Simulations Using Virtual Patient Avatars for Pre-Registration Pharmacist Training: A Mixed Methods Evaluation. Ph.D. Thesis.

[27] Thompson J., White S., Chapman S. (2019). Validation of a questionnaire to evaluate the design of interactive clinical avatars for pre-registration pharmacist training. J. Med. Internet Res..

[28] Shadish W.R., Clark M.H., Steiner P.M. (2008). Can nonrandomized experiments yield accurate answers? A randomized experiment comparing random and nonrandom assignments. J. Am. Stat. Assoc..

[29] Wilkinson S. (2000). Women with breast cancer talking causes: Comparing content, biographical and discursive analyses. Fem. Psychol..

[30] Elo S., Kyngäs H. (2008). The qualitative content analysis process. J. Adv. Nurs..

[31] Royal Pharmaceutical Council (RPS) (2015). Transforming the Pharmacy Workforce in Great Britain: The RPS Vision. https://www.rpharms.com/Portals/0/RPS%20document%20library/Open%20access/Support/Workforce%20and%20Education/transforming-the-pharmacy-workforce-in-great-britain.pdf.

[32] NHS England (2016). General Practice Forward View. https://www.england.nhs.uk/wp-content/uploads/2016/04/gpfv.pdf.

[33] Rosenthal M., Austin Z., Tsuyuki R.T. (2010). Are pharmacists the ultimate barrier to pharmacy practice change?. Can. Pharm. J..

[34] Miller G.E. (1990). The assessment of clinical skills/competence/performance. Acad. Med..

[35] Kolb A.Y., Kolb D.A. (2005). Learning styles and learning spaces: Enhancing experiential learning in higher education. Acad. Learn. Educ..

[36] McGaghie W.C., Issenberg S.B., Petrusa E.R., Scalese R.J. (2010). A critical review of simulation-based medical education research: 2003–2009. Med. Educ..

[37] Ellaway R., Poulton T., Fors U., McGee J.B., Albright S. (2008). Building a virtual patient commons. Med. Teach..

[38] Kelley K.W., Fowlin J.M., Tawfik A.A., Anderson M.C. (2019). The role of using formative assessments in problem-based learning: A health sciences education perspective. Interdiscip. J. Probl. Based Learn..

[39] Salinitri F.D., O’Connell M.B., Garwood C.L., Lehr V.T., Abdallah K. (2012). An Objective Structured Clinical Examination to assess problem-based learning. Am. J. Pharm. Educ..

[40] Krathwohl D.R. (2002). A revision of Bloom’s taxonomy: An overview. Theory Pract..

[41] Huang G., Reynolds R., Candler C. (2007). Virtual patient simulation at U.S. and Canadian medical schools. Acad. Med..

[42] Farrell B., Ward N., Jennings B., Jones C., Jorgenson D., Gubbels-Smith A., Dolovich L., Kennie N. (2016). Participation in online continuing education. Int. J. Pharm. Pract..

[43] Hassell K. (2011). GPhC Register Analysis 2011.

