# Supplementary material for: Actual vs. Perceived Competency Development—How Can Virtual Patients Impact Pharmacist Pre-Registration Training?
Source: Pharmacy (Basel). 2020 Aug 5;8(3):138. doi: 10.3390/pharmacy8030138 (PMC7558302; doi:10.3390/pharmacy8030138)
Supplement: Supplementary file 1 [file pharmacy-08-00138-s001.zip › Supplementary Materials (S2-S5).pdf]

Supplementary File 2 – NI case study one

Learning objectives:

- Communicate with a patient requesting EHC in an appropriate, professional manner
- Demonstrate effective understanding of the information that needs to be obtained from a patient to ensure safe provision of EHC
- Demonstrate effective understanding of the main counselling points associated with EHC
- Describe the different options available for EHC
- Identify the reference sources available for more information regarding EHC

A young woman comes into the community pharmacy where you are working as a pharmacist and requests to speak to you about a personal issue.

1. What are the first things you should do when greeting the patient?

2. The patient requests the morning after pill. What questions do you need to ask a woman requesting emergency hormonal contraception to determine if there is an option suitable for them?

3. The only emergency contraception you have in the store is Levonelle. What advice points do you need to cover to ensure the patient has enough information to make a decision about wanting treatment?

4. From questioning you find out the emergency contraception is for the woman who came into the pharmacy. She is 31 years old and had unprotected sex the night before. No contraceptive measures were taken but the woman is on Yasmin but she admits to finishing her pack a fortnight ago and not having started a new pack yet due to stress. Her last period was about a week ago and it was normal. There is no chance she is already pregnant and she has not used emergency contraception in her current cycle. She takes painkillers and indigestion tablets if she needs them but is on no other medication. She has no liver or malabsorption problems. She has no allergies. Would you supply Levonelle to this patient?

   Yes / No Option

Supplementary File 2 – NI case study two

Learning objectives:

- Practice calculating renal function
- Demonstrate an improvement in calculation skills
- Explain the various ways in which renal function can affect drug doses
- Practice modifying drug dosages accurately
- Identify the reference sources available for more information regarding renal function

Mr Foiles, a 69 year old male has been admitted to hospital following a fall in the night.

His hospital notes and medication chart can be accessed using the link below:

https://docs.google.com/a/keele.ac.uk/file/d/0B51vLJipmVOIR2trQjlxLXN4NTA/preview

Have a read over his notes and then answer the questions below.

1. What is the patient's CrCl?

2. Do any drugs need to be adjusted based on the patient's renal function?

3. Are there any other issues with the patient's medication?

Supplementary File 2 – NI case study three

Learning objectives:

- Demonstrate an improvement in knowledge of common childhood conditions
- Describe the differences between several childhood conditions
- Explain the options available for OTC treatment in children
- Identify the reference sources available for more information regarding childhood conditions

A mother and a child come into the pharmacy. The boy has developed a rash and the mother is wanting information about what it is and what treatment can be provided.

1. What questions do you need to ask to establish what may be wrong with the child?

You find out the child is 5 years old. He has had the rash for ~2 days and it started behind his ears and face as a red and blotchy rash and has now moved down his neck and onto his arms and legs. Before the rash appeared the child had a cold; a bad temperature and a cough which keeps him awake at night. After looking, you notice some white spots in the child's mouth. Upon further questioning you find out the child hasn't been around anybody else with a rash, has had his MMR vaccine and has already had chicken pox. He hasn't been abroad anywhere recently and hasn't got any allergies. The patient is on no other medication and no previous treatment has been tried.

2. What do you think may be wrong with the child?

3. What treatment advice would you give to the mother?

Supplementary File 3 - MCQ quiz for case one

1. How long is Levonelle licensed to be taken after unprotected intercourse?
   a) 24 hours
   b) 48 hours
   c) 72 hours
   d) 96 hours
   e) 120 hours

2. How long is EllaOne licensed to be taken after unprotected intercourse?
   a) 24 hours
   b) 48 hours
   c) 72 hours
   d) 96 hours
   e) 120 hours

3. Which of these medicines may affect absorption of Levonelle?
   a) Carbamazepine
   b) Ciclosporin
   c) Ciprofloxacin
   d) Phenobarbital
   e) Erythromycin

4. How old must a patient be for a sale of Levonelle?
   a) 14
   b) 15
   c) 16
   d) 17
   e) 18

5. Which of the following may prevent a patient having EllaOne?
   a) Lactose intolerance
   b) Crohn's disease
   c) Asthma not controlled by glucocorticoids
   d) Coeliac disease
   e) Rifampacin for tuberculosis

6. Which of the following may prevent a patient having Levonelle?
   a) Lactose intolerance
   b) Crohn's disease
   c) Asthma not controlled by glucocorticoids
   d) Coeliac disease
   e) Rifampacin for tuberculosis

7. What is the effectiveness of EllaOne at preventing pregnancy if taken 120 hours after unprotected intercourse?
    a) 98%
    b) 84%
    c) 75%
    d) 68%
    e) 56%

8. What is the effectiveness of Levonelle at preventing pregnancy if taken 24 hours after unprotected intercourse?
    a) 100%
    b) 98%
    c) 95%
    d) 90%
    e) 85%

9. What type of intrauterine device is available as emergency contraception?
    a) Copper
    b) Titanium
    c) Hormonal
    d) Silicone
    e) Aluminium

10. How long can the emergency contraceptive intrauterine device be inserted after sexual intercourse?
    a) 24 hours
    b) 48 hours
    c) 72 hours
    d) 96 hours
    e) 120 hours

11. Which of these are key points which need to be covered when counselling a patient requesting emergency contraception?
    a) Risk of sexually transmitted infections
    b) Concomitant antibiotic use
    c) Side effects of treatment
    d) Effectiveness of treatment
    e) Regular contraceptive methods

12. What are the most common side effects associated with Levonelle use?
    a) Nausea
    b) Lower abdominal pain
    c) Heartburn
    d) Dsymennorheoa

    e) Constipation

13. When taking Levonelle for EHC a patient will require a second dose if they vomit within what period of time?
    a) 1 hour
    b) 2 hours
    c) 3 hours
    d) 4 hours
    e) 5 hours

14. When taking EllaOne for EHC a patient will require a second dose if they vomit within what period of time?
    a) 1 hour
    b) 2 hours
    c) 3 hours
    d) 4 hours
    e) 5 hours

15. You can take Levonelle more than once in the same cycle
    a) True
    b) False

16. You can take EllaOne more than once in the same cycle
    a) True
    b) False

17. A patient can take both Levonelle and EllaOne in the same cycle?
    a) True
    b) False

18. Patients can get emergency contraception before unprotected sex has taken place
    a) True
    b) False

19. Patients on cerazette should take emergency contraception if they have taken a tablet more than 3 hours late and have had unprotected sex
    a) True
    b) False

20. Patients allergic to levonorgestrel cannot take EllaOne
    a) True
    b) False

Supplementary File 3 - MCQ quiz for case two

1. What resources are available for information on drug doses and renal function?
   a) BNF
   b) Martindale
   c) Summary of Product Characteristics
   d) NEWT
   e) The Renal Drug Handbook

2. Which of these eGFR values indicates a person with severe (stage 4) renal function?
   a) >90
   b) 60-89
   c) 30-59
   d) 15-29
   e) <15

3. Which of these drugs can cause acute kidney injury?
   a) Metformin
   b) NSAIDs
   c) ACEI
   d) Penicillins
   e) Allopurinol

4. Which of these drugs requires dose adjustment if renal function <30ml/min?
   a) Gentamycin
   b) Tazocin
   c) Phenytoin
   d) Teicoplanin
   e) Paracetamol

5. Which of these may require a LMWH dose to be adjusted?
   a) Hepatic impairment
   b) Renal impairment
   c) Cardiac disease
   d) Body weight
   e) Age

6. Bleeding is the most common side effect from treatment with a LMWH
   a) True
   b) False

7. Heparin treatment may result in hypokalaemia
   a) True
   b) False

May result in hyperkalaemia - Heparin can suppress adrenal secretion of aldosterone leading to hyperkalaemia

8. Heparin induced thrombocytopenia occurs when platelet count is reduced by 20%
   a) True
   b) False
   Occurs when reduced by 30% and usually develops after 5-10 days treatment with a heparin

9. For patients at both extremes of weight eGFR is a more accurate measure of renal function
   a) True
   b) False
   Creatinine clearance should be used in those BMI <18.5 or >30

10. Dosages should not be adjusted based on eGFR renal function calculation in those under 18
    a) True
    b) False
    Should use Cockroft and Gault CrCl

11. A patient has been put on rivaroxaban for VTE prophylaxis after hip replacement surgery at a dose of 10mg OD for 5/52. The patient has already had 2 doses, how many 10mg tablets do they need to be sent home with for a complete course to be taken
    a) 23
    b) 25
    c) 33
    d) 35
    e) 42

12. 200mg of potassium permanganate is dissolved in 2.5L of water, what is the % strength?
    a) 0.004%
    b) 0.008%
    c) 0.016%
    d) 0.08%
    e) 0.16%

13. A patient with atrial fibrillation is started on the usual stabilising regime followed by the usual maintenance dose of amiodarone. How many 200mg amiodarone tablets are required for 2 months treatment?
    a) 38
    b) 49
    c) 52
    d) 77
    e) 98

14. A patient has been on 30mg MST Continus BD and Oramorph 20mg over 24 hours for pain relief. What is the equivalent dose of diamorphine SC over 24 hours?
    a) 4mg
    b) 9mg
    c) 15mg
    d) 18mg
    e) 24mg

15. A patient has been taking 40mg citalopram OD. They are in hospital and are unable to swallow tablets so are being converted to citalopram oral drops. What is the equivalent number of drops per day that the patient should take?
    a) 10
    b) 16
    c) 20
    d) 24
    e) 30

16. A 25 year old female patient requires treatment with Argatroban due to heparin induced thrombocytopenia. This should be given by continuous IV infusion at a dose of 2mcg/kg/min. The patient weighs 56kg. What amount of drug will the patient receive over 1 hour?
    a) 0.112mg
    b) 3.36mg
    c) 6.72mg
    d) 67.2mg
    e) 112mg

17. Fucidin cream contains 2% sodium fusidate. What weight of sodium fusidate is present in 35g Fucidin cream?
    a) 60mg
    b) 70mg
    c) 300mg
    d) 600mg
    e) 700mg

18. How many Rifater tablets are required for the initial phase of unsupervised treatment of TB for a patient weighing 65kg?
    a) 140
    b) 168
    c) 280
    d) 336
    e) 672

19. An adult weighing 48kg requires gentamicin treatment of 3mg/kg/day in 3 divided doses. What volume of 40mg/ml injection should be given every 8 hours?
    a) 0.61ml

b) 1.2ml
c) 1.6ml
d) 3.3ml
e) 3.6ml

20. What weight of NaCl is required to produce 1.5L of a solution, such that 20ml of this solution diluted to 500ml gives a 0.2% w/v solution?
    a) 5g
    b) 25g
    c) 50g
    d) 75g
    e) 100g

Supplementary File 3 - MCQ quiz for case two

1. What is the correct paracetamol dose for a 3 year old child in pain?
    a) 30mg every 4-6 hours
    b) 60mg every 4-6 hours
    c) 120mg every 4-6 hours
    d) 180mg every 4-6 hours
    e) 240mg every 4-6 hours

2. What is the correct dose of ibuprofen for an 11 year old child in pain?
    a) 100mg TDS
    b) 150mg TDS
    c) 200mg TDS
    d) 250mg TDS
    e) 300mg TDS

3. Which of these is a symptom associated with a measles infection?
    a) Red-brown rash
    b) Cold symptoms
    c) Temperature
    d) Productive cough
    e) Swelling of salivary glands

4. What is the name of the white spots that appear in the mouth associated with measles?
    a) Koplik spots
    b) Urticaria
    c) Comedones
    d) Cold sores
    e) Strep throat

5. Which of these symptoms are associated with meningitis?
    a) Dark red-purple rash
    b) Rash which does not blanch
    c) Sensitivity to light
    d) Stiff neck
    e) Vomiting

6. Which of these medicines should not be given to children under 16 years of age?
    a) Aspirin
    b) Codeine
    c) Diclofenac
    d) Tetracyclines
    e) Senna

7. Which childhood disease has symptoms similar to the common cold with difficulty breathing and a barking cough?
    a) Pertussis
    b) Croup
    c) Colic
    d) Strep throat
    e) Influenza

8. Which childhood disease is characterised with an itchy red rash that develops into fluid filled blisters which eventually crust over?
    a) Measles
    b) German measles
    c) Chicken pox
    d) Impetigo
    e) Hand, foot and mouth

9. Which childhood disease is characterised by fever, headache and painful swelling of the salivary glands causing the cheeks to puff out?
    a) Roseola
    b) Rubella
    c) Mumps
    d) Croup
    e) Sinusitis

10. What childhood skin infection is associated with a rash of red bumps or blisters that may ooze or be covered with a honey-coloured crust?
    a) Hand, foot and mouth syndrome
    b) Impetigo
    c) Cold sores
    d) Canker sores
    e) Boils

11. Chicken pox is caused by the herpes zoster virus
    a) True
    b) False

12. Cold sores are caused by the human papilloma virus (HPV)
    a) True
    b) False

13. Colic usually starts after 4 weeks of age
    a) True
    b) False
    (most common under 2 weeks)

14. Children with tonsillitis who are allergic to penicillin should be treated with co-amoxiclav?
    a) True
    b) False

    Co-amoxiclav contains amoxicillin and clavulanic acid therefore a patient with severe penicillin allergy should not receive this. Penicillin allergic patients should not be treated with any penicillins, cephalosporins or other beta-lactam antibiotics (carbapenems and aztreonam) due to structural cross-reactivity.

15. Whooping cough is also known as pertussis
    a) True
    b) False

16. Use of aspirin in under 16's can result in Reye's syndrome?
    a) True
    b) False

17. Chloramphenicol for conjunctivitis can be used over the counter in children over 2 years
    a) True
    b) False

18. Loperamide can be used as an over the counter antidiarrhoeal in children from the age of 6
    a) True
    b) False

    Over the age of 12

19. Fifth disease is characterised by a prominent red rash on the face?
    a) True
    b) False

20. Mebendazole is licensed for the treatment of threadworms in children older than 2 years
    a) True
    b) False

Supplementary File 4: Data Collection Questionnaire

Name: ____________________

Gender: Female ☐ Male ☐

Age: _____

Ethnicity: ___________

Main sector of pre-registration training: Hospital ☐ Community ☐

Region of pre-registration training: ____________________

School of Pharmacy: ____________________

Degree Classification: ________________

Average number of times you accessed the virtual patient cases: ____________

Have you had any previous virtual patient experience? Yes ☐ No ☐

Please tick the box that relates to your agreement with the following statements for the case studies:

	Strongly Disagree	Disagree	Undecided	Agree	Strongly Agree
The case studies provided a realistic patient simulation					
When completing the case studies I felt as if I were the pharmacist caring for this patient					
When completing the case studies I felt I had to make the same decisions as a pharmacist would in real life					
The case studies were interesting					
The case studies were enjoyable					
The difficulty of the case studies were appropriate for my level of training					
The feedback I received was adequate for my needs					
The objectives for the case studies were clear and easy to understand					
I was able to access the case studies at my convenience					
The case studies helped develop my clinical reasoning skills					
The case studies helped develop my problem-solving and decision-making skills					
The case studies have helped me to put theory into practice					
I am confident I am developing skills from the case studies that will be required in practice					

I am confident I am gaining knowledge from the case studies that will be required in practice					
It is my responsibility to learn what I need to know from the case studies					
Completing the case studies has improved my confidence for the pre-registration exam					
I feel better prepared to care for real-life patients					
I feel more confident about collaborating with patients and other healthcare professionals					
The case studies have increased my confidence about practicing as a pharmacist					
Overall, the experience has enhanced my learning					

1. What did you like about the case-studies?

2. What did you dislike about the case studies?

3. Which case studies did you find most useful?

4. Which case studies did you find least useful?

5. How do you think the case studies would best fit into the pre-registration training year?

6. Are there any improvements which could be made to the case studies?

7. Are there any other case study topics you would have found useful?

8. Any additional thoughts or comments:

Supplementary File 5: Content analysis themes and frequencies

Theme	Number of trainees	
	VP Group	NI Group
**Features of the Learning Tools**		
Novelty	24	0
Realism (General)	18	0
Realism (topics)	18	19
Feedback	11	9
Interactivity	10	12
**Ease of use of the learning tools**		
Recognition aspects	16	0
Accessibility	8	4
**Trainee development**		
Skill development	10	11
Knowledge development	16	12
Applying learning to practice	19	3
Use of pre- and post-MCQs	5	13
**Integration of the learning tools**		
Individual revision aid	18	25
Group learning	15	13
Cross-sector development	6	0
Competency development	8	0
